# Associations Between Psycho-Social-Spiritual Interventions, Fewer Aggressive End-of-Life Measures, and Increased Time After Final Oncologic Treatment

**DOI:** 10.1093/oncolo/oyad037

**Published:** 2023-04-10

**Authors:** Michael Schultz, Svetlana Baziliansky, Inbal Mitnik, Nirit Ulitzur, Shay Illouz, Duaa Katra, Simon Givoli, Salvatore Campisi-Pinto, Gil Bar-Sela, Daniela Zalman

**Affiliations:** Division of Oncology, Rambam Health Care Campus, Haifa, Israel; Division of Oncology, Rambam Health Care Campus, Haifa, Israel; Division of Oncology, Rambam Health Care Campus, Haifa, Israel; Division of Oncology, Rambam Health Care Campus, Haifa, Israel; Bruce Rappaport Faculty of Medicine, Technion-Israel Institute of Technology, Haifa, Israel; Bruce Rappaport Faculty of Medicine, Technion-Israel Institute of Technology, Haifa, Israel; Statistical Department, Midot Ltd, Israel; Research Authority, Emek Medical Center, Afula, Israel; Bruce Rappaport Faculty of Medicine, Technion-Israel Institute of Technology, Haifa, Israel; Cancer Center, Emek Medical Center, Afula, Israel; Division of Oncology, Rambam Health Care Campus, Haifa, Israel; Bruce Rappaport Faculty of Medicine, Technion-Israel Institute of Technology, Haifa, Israel

**Keywords:** palliative care, oncology, chaplaincy service, social work, psychology, end-of-life care

## Abstract

**Background:**

Little is known about the impact of spiritual caregivers, psychologists, and social workers on desired end-of-life (EoL) medical outcomes, such as reduced use of aggressive care in the final 2 weeks of life, having more time between the last active oncological treatment and death, and increased hospice use.

**Patients and Methods:**

We conducted a prospective study of 180 patients with cancer and their families, their interactions with social work, psychology, and spiritual care, and the above three treatment outcomes.

**Results:**

We found that having one or more spiritual care visits (adjusted odds ratio (AOR) = 2.02; *P* = .04), having more quality visits with the psychologist (*P* = .01), and speaking with someone about one’s inner resources (AOR = 2.25; *P* = .03) all correlated with reduced EoL aggressive care. The key interventions correlating with increased time after final treatment were more visits with the spiritual caregiver or the social worker (AOR = 1.30; *P* < .001), and speaking about the medical treatment (AOR = 1.54; *P* < .001) and about interpersonal relationships (AOR = 2.28; *P* < .001). A subjectively good-quality connection with the spiritual caregiver correlated with increased hospice use (AOR = 10.00; *P* = .01).

**Conclusions:**

Patients with cancer who availed themselves of the spiritual care, psychology, and social work services, each profession in distinct ways, had significantly different outcomes in their EoL medical treatment, including undergoing fewer futile aggressive measures, having more time after their last active treatment, and using hospice services more. These outcomes directly bear on improved quality of life and reduced costs.

## Teaser

This article examines the extent to which psycho-social-spiritual interventions affect key palliative care outcomes, including reduced aggressive measures undertaken in the final 2 weeks of life, increased time between the last active treatment and end-of-life, and increased use of hospice.

**Implications for Practice**

To improve the quality of life for patients with terminal cancer at the end of their lives, the authors identify interventions that enable those patients and families who, given their family dynamic, history, and approach to life, would prefer to avoid difficult treatments at the very end of life (EoL), to have more time from the last active treatment until death, and to receive hospice care. This study found a novel correlation between specific aspects of spiritual care, psychology, and social work provision and these EoL life treatment decisions, suggesting that they may have an important role to play in facilitating these outcomes.

## Introduction

Patients receiving palliative and supportive care should be able to expect to see, when needed, a social worker, psychologist, or spiritual caregiver.^1,2^ These interventions have been found to promote the WHO palliative care goals to “help patients live as actively as possible until death” and “enhance quality of life” in a number of ways. However, not many studies have examined whether one of the pathways for their contribution toward these goals is by directly impacting key medical palliative outcomes: reduced use of aggressive medical care at the end-of-life (EoL) and increased time from the final oncologic treatment until death.

Aggressive medical care at the very EoL, including intensive care unit (ICU) hospitalization or receiving chemotherapy, entails significant costs for patient well-being without extending life,^3,4^ as well as significant fiscal costs.^5,6^ Living as actively as possible until death can often be furthered by increasing the amount of time from the last chemotherapy until death. Increased involvement of the palliative care team is associated with improving these outcomes,^7,8^ yet those results do not isolate the contribution of the psycho-social-spiritual interventions.

Other pathways by which psychological interventions improve quality of life (QoL) and living as actively as possible for patients with cancer include reducing anxiety, depression, and despair,^9,10^ and improving social functioning.^10^ They have a positive effect on biological variables such as neuroendocrine and immune function indicators^11^ and lowered markers of inflammation^12^ as well as on health outcomes such as performance status and symptomatology^13^ and reduced cancer recurrence, possibly by virtue of reducing emotional distress.^14^ Psychological interventions specifically given to palliative care patients demonstrate the increased will to live and sense of meaning and purpose, improved QoL, and reduced depression and anxiety.^15-17^

Spiritual well-being correlates with reduced despair and the wish to die^18^ and depression^19^ among palliative care patients with cancer. Hospitals providing spiritual care see greater use of hospice.^20^ One noteworthy study found that staff provision of spiritual support correlated with a reduction in aggressive treatments at the EoL, improved QoL scores, and greater use of hospice.^6,21^

Social work interventions for dying patients with cancer,^22^ considering both the patient and the systems of which they are a member, particularly the family system and the patient’s culture,^23^ were found to reduce anxiety, depression, and physical suffering and show an increase in patients’ speaking about and being able to accept their upcoming death.^22,24,25^

This study examined the extent to which psycho-social-­spiritual interventions impacted key palliative care outcomes:

Reduced aggressive measures undertaken in the final 2 weeks of life.
Increased time between the last active treatment and EoL.
Increased use of hospice.

## Materials and Methods

For 15 months (November 2018 to March 2020), the directors of the oncology departments at the study site, a large tertiary care hospital, identified all hospitalized patients meeting the primary inclusion criteria—advanced patients with cancer with a life expectancy under 6 months, using the surprise question (“would it surprise you if the patient died in the next 6 months?”). Of these 562 patients, 199 did not meet the other inclusion criteria: 87 did not have the cognitive or physical capacity needed to complete the questionnaire, 104 did not have basic Hebrew fluency, and 8 were primarily treated elsewhere in the last 2 months of life. Of the remaining 363 patients, 231 (64%) gave their informed consent to enter the study. At the end of the study period (7/2020), 180 of these patients had passed away, comprising the final study sample.

There were no significant differences between those eligible patients who did and did not enter the study, in demographics, type of cancer, the time from diagnosis until death, or the time from diagnosis until referral to the study. Demographic data for the final study sample are found in Table 1.

**Table 1.** Demographic and descriptive data for the study sample.

Characteristics	Data
Gender	
Male	52%
Female	48%
Age, years, mean (SD)	63 (12)
No. of children, mean (SD)	2.8 (1.7)
Family status	
Married	72%
Divorced	16%
Widowed	7%
Single	5%
Religion	
Jewish	72%
Muslim	16%
Christian	6%
Druze	3%
Unspecified	3%
Country of birth	
Israel-Jewish	34%
Israel-Arab	26%
Former USSR	14%
Arab countries	13%
Eastern Europe	7%
Western Europe	2%
Others	2%
Spirituality	
Somewhat spiritual	47%
Very spiritual	29%
Not spiritual	23%
Religiosity	
Not religious	47%
Traditional	40%
Religious	13%
Education level	
High school/professional	45%
Higher education	37%
Elementary only	18%
Economic status, self-reported	
Average	65%
Below average	23%
Above average	13%
Type of cancer	
GI	35%
Lung	27%
Breast	12%
GU	12%
Head and neck	7%
Gynaecological	3%
Melanoma and sarcoma	3%
Neurological	1%
Place of residence	
Home	97%
Supportive housing or nursing facility	2%
Living with partner	
Yes	70%
	Note: of those with a partner, 82% said the partner provides “a lot” or “quite a lot” of support
Belongs to a supportive social group	
Yes	58%
Support of friends and family	
High support level	81%
Medium	12%
Low	7%
Holocaust	
Holocaust survivor	6%
2nd generation	24%
Philosophical view of illness (can select 0 or more options)	
Fate	43%
Part of life’s randomness	27%
A call for change	19%
Punishment	10%
Enemy	4%
Calmness in past week (increasing calmness scores 1-5)	
Mean (SD)	2.6 (1.3)
Not calm (1-2)	41%
Somewhat calm (3)	31%
Calm (4-5)	28%
Preference for preserving QoL vs. aggressive treatment (from 1 to 10, with 1 highest focus on QoL, 10 on aggressive treatment)	
Mean (SD)	6.0 (3.1)
Strong preference QoL (1-3)	27%
Weak preference QoL (4-5)	17%
Weak preference aggressive (6-7)	13%
Strong preference aggressive (8-10)	43%

Abbreviations: GI, gastrointestinal; GU, genitourinary; QoL, quality of life.

All patients, whether enrolled in the study or not, continued to receive the standard care provided by the multidisciplinary team. At the study site, the primary medical staff in the oncology departments includes an attending physician and multiple nurses who are board-certified palliative care specialists. As a result, almost all patients in the study saw a palliative care specialist.

The patient entry questionnaire items included demographics, family and social support, spirituality, religiosity, Holocaust history, Steinhauser measure of calmness, general approach to aggressiveness of care, and philosophical view of illness.

Aggressive measures in the final 2 weeks of life were defined as one or more of the following: ICU hospitalization, 2 or more trips to the emergency department, intubation, resuscitation, chemotherapy or immunological therapy, dialysis, central catheterization, pleural drain, or CPAP/BiPAP. Medical data were collected from the patient chart and were accessible even if patients passed away not at the study site.

The study questions were examined against the following interventions, for each profession separately (for those patients who saw that kind of professional) and for all 3 professions jointly when patients saw more than one kind of professional:

Receiving a visit from that kind of professional (yes/no).
No. of visits.
Intensity of visits (no. of visits/time from metastatic diagnosis until death).
Time from metastatic diagnosis to first visit.
Duration of the therapeutic relationship (time from first visit to last visit).
Subjective quality of connection.
Subjective contribution of visits (no. of visits making a significant contribution).
Content of conversations.
Actions taken in light of the conversations.

Intervention data were collected from the date of metastatic diagnosis using a chart review and a form completed by social workers, spiritual caregivers, and psychologists who met with the patients and their families regarding these interactions. In addition, we reviewed the chart to see whether there was a palliative care conversation with the physician and how long before death it took place. Over the course of the study period, the staff included 8 social workers, 4 psychologists, and 5 spiritual caregivers.

Potential confounders considered included: the above patient questionnaire data, time from first visit to death, time from last visit to death, time from metastatic diagnosis to death, time from study enrollment to death, type of cancer and of cancer treatment, performance status at study enrollment, and for aggressive treatment analysis—number of days hospitalized and percent of time hospitalized.

Spiritual care in this setting takes an approach that understands spirituality broadly. The intent is to understand and engage with patients’ own approach to life, offering patients of any or no religious orientation a means of connecting to their inner, interpersonal, and broader (eg, faith, values, connection to nature) resources to aid in coping with illness and loss.

The study was conducted in accordance with the Declaration of Helsinki, and approved by the Institutional Review Board (or Ethics Committee) of Rambam Health Care Campus (protocol code 0556-17-RMB, approved January 4, 2018).

### Statistical Analysis

For each research question, the statistical analyses were conducted in 3 stages. First, univariate relationships between the dependent variable of each research question and each of the independent measures were explored. Depending on the statistical properties of the variables, Chi-square test of independence (when both variables were nominal), Mann-Whitney test (when one variable was continuous and the other nominal with 2 categories), Kruskal-Wallis test (when one variable was continuous and the other nominal with more than 2 categories), or Spearman’s correlations coefficient (when both variables were continuous) were computed. In the second stage, the univariate relationships between each dependent variable and the confounding variables were examined. In the final stage, multivariate models were constructed: for each significant independent measure found in the first stage (*P* < .05), significant confounders (*P* < .05) found in the second step were added to the model. Specifically, in the aggressive measures and hospice questions, forward stepwise logistic regressions were conducted, and in the time from the last treatment question, a Poisson regression was used.

In all multivariate regressions, the independent measure was added in the first step of the logistic regression, and all confounders were added in the second step. Confounders were entered into the model only if they changed the dependent variable’s parameter estimate by more than 10% and retained a significant *P*-value (*P* < .05), after controlling for other confounds. Odds ratios (OR) with 95% CI were computed for significant models. All statistical analyses were performed using SPSS 25 software for Windows.

## Results

### Receipt of Psycho-Social-Spiritual Care

Of 180 patients, 154 had at least one visit with a social worker, 99 with a spiritual caregiver, and 41 with a psychologist. Some patients saw more than one kind of professional: 84 patients saw both a social worker and a spiritual caregiver, 41 saw both a social worker and a psychologist, and 30 saw both a psychologist and a spiritual caregiver.

There were relatively few correlations between demographics, including medical data, and the study interventions. Women were relatively more likely than men to have more sessions with a psychologist (*P* = .003) and with a spiritual caregiver (*P* = .03). The families of Jewish patients born in Israel were more likely to see a spiritual caregiver (*P* = .02). Older patients were referred to spiritual care sooner after metastatic diagnosis (*P* = .003). Patients who were less calm had more conversations with the social worker (*P* = .01).

### Reduced Aggressive Measures in the EoL

Of 180 patients, 61 (34%) underwent at least one aggressive measure in the final 2 weeks of life, where the most common such measures were chemotherapy (12%), central catheterization (11%), ICU (9%), and pleural drain (8%).

Several potential confounders showed a univariate correlation with this study question: less religiosity, more time from first visit to death, more time from last visit to death, viewing illness as part of life’s randomness, and slower disease progression. None of the other variables significantly correlated, including the patient questionnaire items, notably among them personal approach to aggressiveness of care, and personal spirituality.

Multivariable logistic regression models examined the potential impact of those confounders significant in the univariate analysis, as well as the most hypothetically likely confounders even though they were not significant in the univariate analysis, on the study interventions that significantly correlated with reduced aggressive EoL care. Those interventions that remained significant are presented in Table 2.

**Table 2.** Interventions correlating with reduced aggressive treatments at the EoL, multivariate models.

Intervention	Adjusted OR	95% CI	*P*-value
Spiritual care			
Any spiritual care for the patient	2.02	1.01-4.02	.04
Viewing illness as part of life’s randomness	2.91	1.21-7.00	.02
Slower disease progression (months from enrollment to death)	1.20	1.04-1.40	.02
Inner/transpersonal resources			
Discussing inner or transpersonal resources with any professional	2.25	1.11-4.56	.03
Viewing illness as part of life’s randomness	2.44	1.01-5.89	.04
Slower disease progression	1.19	1.02-1.38	.03
Medical system			
Discussing dealing with the medical system and treatment	0.20 (reverse impact)	0.05-0.72	.01
Viewing illness as part of life’s randomness	2.93	1.20-7.12	.02
Slower disease progression	1.22	1.05-1.42	.01
Palliative care conversation			
Palliative care conversation with physician >1 week before death	4.56	1.86-11.18	.001
Slower disease progression	1.29	1.04-1.60	.02

Variables adjusted for less religiosity, more time from first visit to death, more time from last visit to death, viewing illness as part of life’s randomness, perception of supportive partner or supportive family and friends, general approach to the aggressiveness of care, # of days hospitalized, and slower disease progression.

Abbreviations: End-of-Life, EoL; OR, odds ratio.

Both discussing one’s past with the psychologist (*P* = .03) and the # of sessions with the psychologist that made a clear contribution to patient well-being (*P* = .01) had significant univariate correlations with reduced aggressive EoL care, but because of their small *N* we did not include them in the multivariate analysis. The # of visits the spiritual caregiver had with family members maintained a strong positive correlation with this study goal when comparing 1-2 vs. 3 or more such visits (*P* = .02).

### Increased Time Between Final Active Treatment and Death

On average, patients had 62.7 days from their last active treatment until death (median 37.5 days; SD 82.3).

Adjusted models for the most significant interventions correlating with a longer period of time between the last treatment and death are presented in Table 3. These interventions include speaking about interpersonal relationships with the psychologist (*β* = 0.82); speaking with the spiritual caregiver about dealing with the medical system and treatment (*β* = 0.43); the # of spiritual care visits with the family that clearly made a contribution (*β* = 0.26); the total # of visits with the patients’ family members (spiritual care (*β* = 0.09), social work (*β* = 0.02)); and having a palliative care conversation with the physician at least one month before the patient’s death (*β* = 0.53).

**Table 3.** Select key interventions correlating with increased time from final active treatment until death, multivariate analysis.

Intervention	Adjusted OR	95% CI	*P*-value
Interpersonal relationships			
Discussing interpersonal relationships with the psychologist	2.28	1.94-2.68	<.001
Medical system			
Discussing dealing with the medical system and treatment with the spiritual caregiver	1.54	1.42-1.66	<.001
More time from first visit with patient until death	1.02	1.01-1.02	<.001
More time from diagnosis to death	0.99	0.99-1.00	<.001
Slower disease progression	1.02	1.01-1.03	<.001
Significant spiritual care visits			
# Spiritual care visits with family making a clear subjective contribution	1.30	1.25-1.35	<.001
More time from first visit with family until death	1.64	1.43-1.87	<.001
Slower disease progression	1.17	1.14-1.19	<.001
More time from first visit with patient until death	0.60	0.53-0.69	<.001
Greater patient age	0.99	0.98-0.99	<.001
More time from diagnosis to death	1.01	1.01-1.02	<.001
Social work visits			
# Social work visits with family	1.02	1.02-1.03	<.001
Departmental affiliation	0.89	0.87-0.91	<.001
Slower disease progression	1.06	1.05-1.07	<.001
More time from first visit with family until death	1.01	1.01-1.02	<.001
Greater patient age	1.01	1.00-1.01	<.001
More time from diagnosis to death	0.996	0.995-0.997	<.001
Spiritual care visits			
# Spiritual care visits with family	1.09	1.07-1.11	<.001
More time from first visit with family until death	1.16	1.13-1.20	<.001
More time from first visit with patients until death	0.83	0.80-0.87	<.001
Slower disease progression	1.13	1.11-1.15	<.001
Greater patient age	0.99	0.99-1.00	<.001
Palliative care conversation			
Palliative care conversation with physician > 1 month before death	1.70	1.60-1.81	<.001
Slower disease progression	1.04	1.03-1.05	<.001
More time from diagnosis to death	1.001	1.000-1.003	.03

Variables adjusted for: time from first visit until death, pace of disease progression, time from diagnosis to death, and age.

### Increased Use of Hospice

Of 155 patients for whom we had sufficient information about hospice use, 78 received hospice while 77 did not. For the 67 patients referred directly to hospice at discharge, the average duration of receipt of hospice services, from discharge to death, was 73.6 days, and the median was 24 days.

In the multivariate analysis, the quality of the connection between spiritual caregiver and family members (*P* = .01, AOR = 10.00, 95% CI, 1.64-60.92), as well as the subjectively rated quality of the connection between all 3 professionals taken together and the patient (*P* = .03, AOR = 4.17, 95% CI, 1.16-14.98), correlated with increased enrollment in hospice. None of the other interventions showed a correlation with this study outcome.

## Discussion

### Reduced Aggressive Treatments at the EoL

Perhaps the most significant novel finding of this study is the correlation between the receipt of professional spiritual care and reduced aggressive EoL treatments. This builds on a previous finding regarding patients feeling spiritually supported by the staff as a whole.^21^ In our study, there was a strong, persistent relationship between the interventions of the spiritual caregiver specifically and reduced aggressive EoL treatments.

Our results highlight spiritual care in general and thinking about one’s inner resources or resources between the patients and that which is greater than them in particular (such as faith, calmness, meaning of life, key values, hope, and prayer; this was the most common content area item in spiritual care visits). We can theorize that spiritual care promotes relative calm and reduces anxiety and distress, similar to what has been found elsewhere among patients with cancer with higher spiritual well-being.^26-32^ Other studies have found that spiritual care interventions reduce family worry and improve coping.^33,34^ These effects, in turn, help enable those families who wish to avoid unhelpful treatments that worsen QoL at the EoL to do so, rather than anxiously asking for or agreeing to these treatments.

Similarly, in light of our findings about the positive impact of psychological interventions, particularly when it involved discussing patients’ pasts with them, it is worth noting specialized EoL psychological interventions that include a strong element of life review and considering one’s legacy, such as dignity therapy and meaning-centered psychotherapy, which have been found to reduce anxiety and improve spiritual well-being.^15-17^

For certain patients or families, there are other factors at play in the decision to undergo aggressive care, such as personal history, approach to life, a desire to do so on behalf of the family, and religiosity and a belief in miracles, and these factors may outweigh the desire to maximize QoL.^35-37^ In those cases, the palliative team will still support the patients in their approach.

Our study, by asking about patients’ philosophical approach to illness, may help identify a positive belief that is associated with improved palliative care outcomes: viewing illness as part of the randomness of life. Other studies found improved prognostic understanding among patients with lower levels of certain religious beliefs, such as a belief in miracles.^37,38^

Regarding the prevalence of aggressive EoL treatments, this study had similar results to previous studies.^21,39,40^ The significant impact of the physician conducting a palliative care conversation relatively early, and not only at the very EoL, as affirmed by our study, is well-established.^40,41^

The association between speaking about dealing with the medical system and increased aggressive care likely reflects those patients whose approach is more interventional, and who may have been experiencing the frustration that this approach was not improving medical outcomes.

The most significant non-interventional factor was the time from when the patient entered the study until death. These patients were not identified as being in their final 6 months of life until they were much closer to death; ie, their condition deteriorated more rapidly. As a result, they were more likely to choose to undergo aggressive treatments, since they had had less time to internalize that the EoL was rapidly approaching.

Though the study design included in the statistical analysis all the relevant potential confounding factors we could identify, we should still note that these results show associations rather than causation. It is possible that other factors led to both an increased use of a particular intervention and to the study outcomes.

### Increased Time From Last Treatment Until Death

Continuing to receive active oncologic treatments at the EoL, in particular, chemotherapy, does not improve overall QoL near death and even worsens it.^42^ Lengthening the duration of the period after the last active treatment is a valuable palliative care goal, enabling the patient to have a period of time at the EoL that does not involve dealing with the side effects of treatment.

As expected, physician-patient palliative care conversations improved this outcome in our study, and the earlier the better. At baseline, prognostic understanding is poor.^43^ These conversations help patients and family members understand their situation and make their decisions accordingly, and many patients choose the approach that will maximize their QoL at the EoL.

Though EoL conversations have become more of a standard practice for which physicians received specialized training, recent studies have identified an additional factor necessary for them to translate into a longer period of time between treatment and death. In addition to conveying medical information, the care team needs to help patients and family members be able to emotionally process the information^44^—otherwise, emotional distress impedes their ability to process the information they receive.

In this way, we can understand the significance of the ­psycho-social-spiritual interventions that showed a persistent correlation in our study with a longer period of time after the last treatment, including the total number of visits and the number of clearly valuable visits. We theorize that these conversations, especially when there were more of them over a period of time, helped ameliorate patients’ and family members’ emotional distress, leaving them more able to process their situation and decide accordingly on the care plan moving forward.

In this case, speaking about the medical treatment with the spiritual caregiver and speaking about significant relationships with the psychologist was quite strongly positively associated with the palliative care outcome. We can hypothesize that patients and family members were sharing their inner debate as to whether to continue with treatment or whether the time had come to stop, including potential differences of opinion between patients and families, and for those patients who were inclined to stop, these conversations helped the family unit act on that desire, despite it being such an emotionally fraught decision.

It is worth noting that for patients who stop active treatment earlier, there are fewer opportunities for the social workers and spiritual caregivers to see them, in turn reducing the potential number of visits. Yet the association between these interventions and this palliative care outcome was so strong that it persisted despite these patients having fewer hospital stays.

Another possible explanation for the increased time between the last treatment and death is not just that these patients are stopping treatment earlier, but also that they are living somewhat longer. Palliative care as a whole has been found to lengthen life span,^45^ and the improved emotional and spiritual state may contribute to appetite, activity level, and other factors that contribute to this outcome.

Professional spiritual care provision, as well as other spiritual interventions, specifically correlate with improved QoL at the EoL,^21,27^ as does spiritual well-being at all stages of illness progression,^29,30,32,46,47^ where the impact relates to differences in spirituality rather than in religiosity.^30,31^ There are presumably a number of pathways through which spiritual well-being promotes QoL at the EoL, and stopping active treatments earlier on may be one of them.

### Increased Use of Hospice

One previous study noted that in hospitals where spiritual care is provided, more patients used hospice, but it did not specifically examine which patients received spiritual care.^20^ Another study found that patients who felt their spiritual needs were met by the medical team as a whole were more likely to use hospice, but just looking at whether or not those patients saw the spiritual caregiver they did not see an impact on hospice usage.^21^ That finding was replicated in our study, but by examining the question at a higher resolution—looking not just at whether the patient and family members received spiritual care, but the quality of the connection—the positive association became clear.

### Limitations

The sample size of psychologist interventions was too small to identify moderate, only substantial correlations. Though we identified many potential confounders, including all factors found to be significant in previous studies we carried out at our hospital,^48,49^ we were not able to consider other potential confounders, such as the extent to which patients who are positively inclined to receive the study interventions are already more likely to take a more palliative approach; history of close family members dying of cancer and the medical approach taken at the end of their lives; and patients’ cognitive abilities to engage in an extended conversation.

## Conclusion

This study potentially demonstrates the direct impact of social workers, spiritual caregivers, and psychologists on patients’ palliative medical outcomes, beyond generally reducing suffering and improving QoL. We can now state more clearly some of the specific ways in which these non-medical interventions improve patients’ QoL as a result of improved palliative medical outcomes.

As always, funding is limited, and this study indicates that it is a worthwhile investment to ensure that both spiritual caregivers and psychologists, together with social workers, are on staff in all oncology departments, and by implication also in home hospice, to reduce the unnecessary use of aggressive measures at the EoL. This more than pays for itself in reduced healthcare costs.^6^

This hypothesis-generating study suggests further study specifically of the presence and # of visits, the # of clearly contributing visits, the quality of the connection, the content areas of the visits, and of the interaction between these factors.

## Data Availability

The data underlying this article will be shared on reasonable request to the corresponding author.
